# Research on Rapid and Non-Destructive Detection of Coffee Powder Adulteration Based on Portable Near-Infrared Spectroscopy Technology

**DOI:** 10.3390/foods14030536

**Published:** 2025-02-06

**Authors:** Fujie Zhang, Xiaoning Yu, Lixia Li, Wanxia Song, Defeng Dong, Xiaoxian Yue, Shenao Chen, Qingyu Zeng

**Affiliations:** Faculty of Modern Agricultural Engineering, Kunming University of Science and Technology, Kunming 650500, China; 20030031@kust.edu.cn (F.Z.); 15138169516@sohu.com (X.Y.); songwanxia0721@163.com (W.S.); 15239738900@sohu.com (D.D.); 15171723513@163.com (X.Y.); 13358078882@163.com (S.C.); zqy18287495726@163.com (Q.Z.)

**Keywords:** invasive weed optimization, adulteration identification, qualitative and quantitative detection, feature selection, machine learning

## Abstract

This study explores the feasibility of using portable near-infrared spectroscopy for the rapid and non-destructive detection of coffee adulteration. Spectral data from adulterated coffee samples in the 900–1700 nm range were collected and processed using five preprocessing methods. For qualitative detection, the Support Vector Machine (SVM) algorithm was applied. For quantitative detection, two optimization algorithms, Invasive Weed Optimization (IWO) and Binary Chimp Optimization Algorithm (BChOA), were used for the feature wavelength selection. The results showed that convolution smoothing combined with multiple scattering correction effectively improved the signal-to-noise ratio. SVM achieved 96.88% accuracy for qualitative detection. For the quantitative analysis, the IWO algorithm identified key wavelengths, reducing data dimensionality by 82.46% and improving accuracy by 10.96%, reaching 92.25% accuracy. In conclusion, portable near-infrared spectroscopy technology can be used for the rapid and non-destructive qualitative and quantitative detection of coffee adulteration and can serve as a foundation for the further development of rapid, non-destructive testing devices. At the same time, this method has broad application potential and can be extended to various food products such as dairy, juice, grains, and meat for quality control, traceability, and adulteration detection. Through the feature wavelength selection method, it can effectively identify and extract spectral features associated with these food components (such as fat, protein, or characteristic compounds), thereby improving the accuracy and efficiency of detection, further ensuring food safety and enhancing the level of food quality control.

## 1. Introduction

In recent years, food adulteration has become an increasingly prevalent issue, where unscrupulous merchants add inexpensive materials to food products in a manner that is difficult for consumers to detect, with the aim of gaining higher profits [1]. High-value products are often the primary targets of adulteration. Coffee, a beverage made from roasted and ground coffee beans, is highly popular due to its unique flavor and stimulating effects. It is currently one of the best-selling drinks worldwide [2]. According to data from the International Coffee Organization, global coffee production has recently reached approximately 172 million bags (60 kg/bag) [3]. According to the “2024 China Imported Coffee Industry Report”, the scale of China’s coffee market will have increased by CNY 313.3 billion in 2024 [4], highlighting the significant economic value of coffee, which makes it highly susceptible to adulteration. Taking Arabica coffee beans as an example, their price is approximately CNY 225 per kilogram, while the cost of adulterants is much lower; for example, corn costs only CNY 8 per kilogram. Illegal traders profit greatly by mixing in low-cost materials, causing significant economic losses to the coffee industry. After roasting and grinding, the physical characteristics of coffee beans, such as particle size and color, can be easily mimicked by low-cost materials (such as soybeans, corn, barley, chicory, and brown sugar), making it difficult to detect adulteration [5]. Although some studies have investigated the methods for detecting adulteration in ground coffee, these methods are generally based on techniques such as chromatography [6], mass spectrometry [7], electronic tongues [8], and DNA analysis [9]. These methods, however, tend to be complex, time-consuming, or limited by the high cost of instruments, which makes them impractical for routine analysis. Therefore, there is a pressing need to develop a rapid, non-destructive detection method. Munyendo et al. [10] showed that spectroscopic techniques are rapid, non-destructive, and easily integrable with other processes, possessing great potential for widespread applications. Fourier-transform infrared spectroscopy (FTIR), Raman spectroscopy, fluorescence spectroscopy, and other methods have been widely applied in coffee adulteration detection, quality evaluation, and component analysis, achieving significant progress. While these methods have certain advantages in component analysis, near-infrared (NIR) spectroscopy, by comparison, offers faster detection speed, lower equipment costs, and is more suitable for large-scale, on-site applications.

Near-infrared (NIR) spectroscopy is a form of electromagnetic spectrum, with wavelengths ranging from 780 nm to 2526 nm [11]. The stretching and bending vibrations of each chemical bond (such as C-H, O-H, N-H) have a specific vibration frequency (wave number), so they absorb light energy in a specific wavelength range of the near-infrared spectrum. When near-infrared light is directed onto a sample, the light waves interact with the hydrogen-containing groups in the molecules of the sample. If the frequency of the light matches the vibrational frequencies of these groups, the sample will absorb light at that frequency [12]. Therefore, the NIR spectral characteristics differ depending on the chemical composition of the substance [13]. NIR spectroscopy leverages this principle to analyze the spectral data of a sample, enabling the identification of compositional differences. This technique has been widely applied in various fields of food adulteration detection. For instance, Antoine et al. [14] developed a model for detecting peanut powder adulteration in cocoa powder by collecting NIR spectral data and applying chemometrics, yielding promising results. Khamsopha et al. [15] used NIR spectroscopy combined with Partial Least Squares Regression (PLSR) to develop a model for detecting cassava starch adulteration, with a prediction set correlation coefficient (R^2^) of 0.996. Zhang et al. [16] performed the quantitative detection of adulteration in Sanqi powder by analyzing NIR spectral data, achieving a prediction set correlation coefficient (R^2^) of 0.9667 using a Support Vector Regression (SVR) model. Guo Wenchuan et al. [17] utilized NIR spectroscopy combined with a random forest model to detect adulterated camellia seed oil, achieving an accuracy rate of 99.34%. These studies have demonstrated the feasibility of using NIR spectroscopy for food adulteration detection. However, there is limited research on the rapid, non-destructive detection of coffee adulteration using portable NIR spectroscopy technology.

To address the above-mentioned issue, this study explores the rapid, non-destructive detection capability of coffee adulteration using portable near-infrared (NIR) spectroscopy. The impact of various pre-processing methods on spectral curves and modeling accuracy was compared. Additionally, the effectiveness of two novel intelligent optimization algorithms in selecting key wavelengths was evaluated. The information represented by the selected key wavelengths was analyzed, and its influence on the modeling results was assessed. The findings aim to provide a theoretical basis for the further development of portable detection devices for coffee adulteration.

## 2. Materials and Methods

### 2.1. Sample Preparation

The coffee beans selected for this study were of the Caturra variety (medium roast), belonging to the Arabica species, which is a small-bean coffee. The beans were sourced from Pu’er City, Yunnan Province, China, and have a market price of CNY 225 per kilogram. Due to the difficulty in visually distinguishing adulterants such as soybeans, barley, chicory, and corn after they have been roasted and ground into powder, these materials are commonly used in coffee adulteration. Therefore, they were chosen as adulterants for this study. Basic information on the four adulterants and their roasting methods is shown in Table 1.

The coffee beans, along with the roasted soybean, barley, chicory, or corn, were ground into powder, sift through a 200-mesh sieve to ensure consistent particle size, then placed into sealed bags and stored in a dry, room temperature environment (15 °C) away from direct sunlight. In this study, 20 blank samples were prepared for each of the five materials, resulting in a total of 100 samples for the spectral analysis. Additionally, adulterated samples were prepared by mixing soybean, barley, chicory, and corn with coffee at four different adulteration levels—10%, 20%, 30%, and 40%. This resulted in 16 groups of samples with different types of adulteration. Each group contained 50 samples, each weighing 10 g. The samples were then sealed in bags and labeled, yielding a total of 800 coffee adulteration samples, which were also stored in a dry, room temperature environment (15 °C) away from direct sunlight. These 800 adulterated samples were categorized into four groups based on the type of adulterant, with each group containing 200 samples for qualitative analysis. Furthermore, they were divided into 16 groups based on both the type and proportion of adulterant for both the qualitative and quantitative analysis.

### 2.2. Experimental Instruments

A portable near-infrared spectrometer (YCNIR-1, Yunnan Xiaobao Technology Co., Ltd., Shenzhen, China) was used for data acquisition of the 100 blank samples and 800 adulterated samples. The device can detect spectral wavelengths ranging from 900 to 1700 nm, with a spectral resolution of 10.53 nm, and an exposure time of 2.54 ms during data collection; the average number of scans is 3, and the number of wavelength points is 228. A multifunctional grinder (Model: 1500A, Yongkang Hongtai Electromechanical Co., Ltd., Jinhua, China) was used for sample preparation, and a Leqi electronic balance (accuracy: 0.01 g, Kunshan Youkewite Electronics Technology Co., Ltd., Kunshan, China) was employed for sample weighing.

### 2.3. Spectral Data Collection

The spectral data of the adulterated samples were collected according to the numbered labels on the sealed bags. The samples were transferred into PP sample trays (Polypropylene Reagent Trays), ensuring a smooth surface for spectral collection. Before scanning the spectral data, the portable near-infrared spectrometer was preheated for 15 min, and the ambient temperature in the laboratory was maintained at 25 °C. It was then connected to a mobile phone via Bluetooth, and the data were collected from the samples. In order to reduce errors, each sample was scanned three times, and the average spectrum was taken as the original spectral data. The acquired spectral data were subsequently transferred from the mobile phone to a computer via USB for further modeling and analysis.

### 2.4. Data Processing Methods

#### 2.4.1. Spectral Data Preprocessing

During the spectral data collection process, the instrument may be affected by noise, background light, environmental conditions, and other factors, leading to baseline drift, scattering, and other phenomena. Therefore, it is necessary to preprocess the raw spectral data to enhance the signal-to-noise ratio [18]. In this study, the following preprocessing methods were applied to the raw spectral data of the coffee adulteration samples: Savitzky–Golay smoothing (SG), Standard Normal Variate Transformation (SNV), Multiplicative Scatter Correction (MSC), and two combinations of preprocessing methods (SG-SNV, SG-MSC). The process was carried out using the Unscrambler X 10.4 (64-bit) software, which is a versatile statistical analysis and modeling tool suitable for preprocessing high-dimensional and complex spectral data to help improve data quality.

#### 2.4.2. Spectral Data Dimensionality Reduction

Near-infrared spectral data typically have high dimensionality, containing a large amount of redundant information that does not directly contribute to the chemical characterization of the samples, as well as high correlations between the different wavelengths. These factors can negatively impact the accuracy of the model [19]. Dimensionality reduction of the spectral data can simplify the data structure, eliminate redundant information and noise, and retain the characteristic wavelengths that can best distinguish the differences between the samples, thereby improving the generalization and performance of the model. In this study, two novel intelligent algorithms were employed for the feature wavelength extraction from the coffee adulteration spectral data, namely the Invasive Weed Optimization (IWO) algorithm and the Binary Chimp Optimization Algorithm (BChOA).

The Invasive Weed Optimization (IWO) [20] algorithm is a population-based intelligent optimization algorithm that mimics the process of weeds finding suitable locations for growth. In the feature wavelength selection for the full spectrum of near-infrared spectroscopy data, the Classification Error Rate (CER) is used as a fitness function. The process begins by initializing a weed population and calculating its fitness values, setting a threshold to determine whether the feature wavelength corresponding to a variable will be selected. Weeds randomly propagate in the search space based on a normal distribution, and new subsets of variables are generated according to their fitness values. Weeds with better fitness continue to survive and reproduce, while those with poorer fitness are eliminated. The algorithm iterates to update the optimal variable combination until the maximum number of iterations is reached, ultimately identifying the best feature subset, that is, the optimal combination of feature variables.

The Binary Chimpanzee Optimization (BChOA) [21] algorithm is a feature selection method based on the collective behavior of chimpanzee groups. During feature wavelength selection, the algorithm randomly divides the population into four groups—breakthrough, blockade, pursuit, and attack—to simulate the role division during chimpanzee hunting. The fitness function values of the initial feature variables are then calculated. A transfer function is used to map the continuous search space to a binary space, allowing the chimpanzees to search for feature variables within the binary environment. In each iteration, the variable subsets are evaluated based on the fitness function CER, and the optimal variable set and positions are updated until the iteration ends. The best feature subset obtained at this point is the optimal combination of feature variables.

#### 2.4.3. Detection Model Construction

After preprocessing the raw spectral data, three classification algorithms were chosen to model the raw and preprocessed spectral data, namely Support Vector Machine (SVM), Backpropagation Neural Network (BP), and Random Forest (RF). In this study, the training set and prediction set were randomly divided in an 8:2 ratio, so the number of samples in the training set was 640, and the number of samples in the prediction set was 160. For the qualitative detection of coffee adulteration, the SVM algorithm was selected as the discriminative model. For the more complex task of both the qualitative and quantitative detection of coffee adulteration, the SVM, BP, and RF algorithms were used for model construction, with the best model being selected through comparison. SVM uses cross-validation to adjust key parameters C and gamma. The values selected in this study were C: [1, 10, 100, 1000] and gamma: [0.001, 0.01, 0.1, 1].

#### 2.4.4. Model Evaluation Metric

In this study, the evaluation metrics used to assess the performance of the models are accuracy, precision, and specificity. These metrics are calculated using four indicators from the confusion matrix, as follows: True Positives (*TP*s): The number of samples correctly predicted as belonging to the current class; False Positives (*FP*s): The number of samples incorrectly predicted as belonging to the current class; False Negatives (*FN*s): The number of samples that actually belong to the current class but are not correctly identified by the model; True Negatives (*TN*s): The number of samples correctly identified as belonging to other classes [22]. The calculation formulas for the above indicators are as follows (1)–(3):

(1) $$Accurary=\frac{TP+TN}{TP+TN+FP+FN}$$

(2) $$Precision=\frac{TP}{TP+FP}$$

(3) $$Specificity=\frac{TN}{TN+FP}$$

Accuracy measures the proportion of samples that are correctly classified by the model, reflecting the overall discriminative ability of the model. Precision evaluates the proportion of correctly predicted positive samples among those predicted as positive, reflecting the model’s overall prediction capability. Specificity assesses the proportion of correctly classified negative samples, reflecting the model’s ability to distinguish negative classes.

## 3. Results and Analysis

### 3.1. Spectral Analysis of Samples

**Figure 1:**
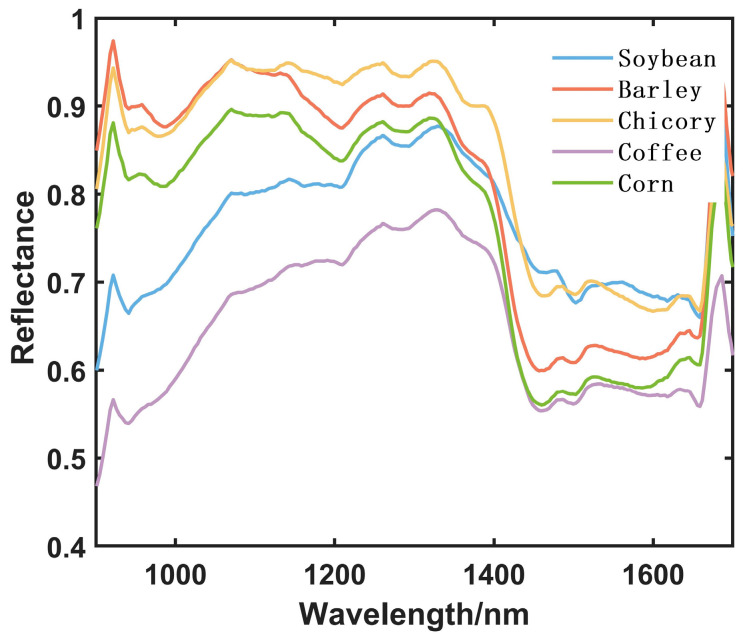
Average Spectral Curves of Soybean (blue solid line), Barley (red solid line), Chicory (yellow solid line), Coffee (purple solid line), and Corn (green solid line).

Near-infrared (NIR) spectroscopy operates by absorbing electromagnetic radiation, which excites specific chemical bonds (such as C-H, N-H, O-H) within molecules, resulting in characteristic absorption peaks [23]. The types and quantities of organic compounds in different substances vary significantly, leading to different absorbances or reflectances in the near-infrared spectral range [24]. In this study, the reflectance spectra of the samples were analyzed to enable the rapid, non-destructive detection of coffee adulteration. After roasting and grinding, soybeans, barley, chicory, and corn exhibit physical properties similar to those of coffee, making it difficult to visually distinguish between them. However, their inherent chemical compositions differ significantly. Soybeans are primarily composed of proteins and fats [25]; barley is rich in carbohydrates and proteins; chicory contains high levels of carotenoids and vitamins [26]; and corn is composed mainly of carbohydrates (primarily starch), dietary fibers, and vitamins. These nutrients have relatively simple chemical structures, typically consisting of carbon, hydrogen, and oxygen, resulting in weak absorption and high reflectance in the near-infrared spectral range. In contrast, coffee contains compounds such as caffeine, chlorogenic acid, and trigonelline [27], and roasted coffee also contains certain amounts of oils, which exhibit strong absorption in the near-infrared spectrum. As a result, the near-infrared spectral curves of soybeans, barley, chicory, corn, and coffee show distinct differences. The average spectral curves of the five materials are shown in Figure 1 below, where the significant differences are evident, particularly at wavelengths of 920 nm, 945 nm, 1070 nm, 1210 nm, 1257 nm, 1462 nm, 1660 nm, and 1686 nm. These differences are mainly observed in the 1400 nm to 1650 nm range, which corresponds to the absorption caused by the O-H vibrations of water molecules.

### 3.2. Data Preprocessing

**Figure 2:**
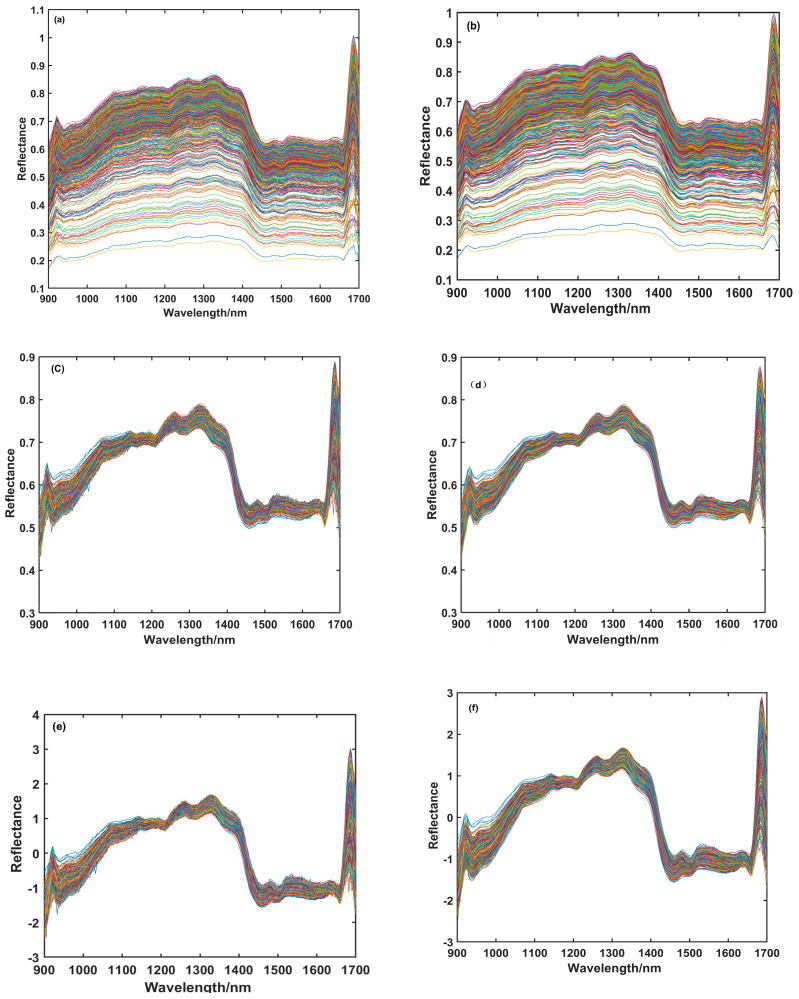
(**a**) Original; (**b**) SG preprocessing; (**c**) MSC preprocessing; (**d**) SG-MSC preprocessing; (**e**) SNV preprocessing; (**f**) SG-SNV preprocessing.

In this study, five preprocessing methods—SG, SNV, MSC, SG-MSC, and SG-SNV—were applied to preprocess the raw spectral data of adulterated coffee. The raw spectral curves and the preprocessed spectral curves are shown in Figure 2.

By comparing Figure 2, it is evident that the raw near-infrared spectral curve (a) is significantly different from the preprocessed near-infrared spectral curve. The spectral curve after SG preprocessing (b) is much smoother, and noise spikes are notably reduced [18]. The spectral curve after MSC preprocessing (c) and the spectral curve after SNV preprocessing (e) are more focused, with a noticeable reduction in the spacing between spectral lines, indicating that these two preprocessing methods significantly improve the scattering and spectral drift phenomena in the near-infrared spectral data acquisition process. Considering the different effects of SG, SNV, and MSC, a combination of two methods is chosen to improve the signal-to-noise ratio of the raw spectral data [28]. The spectral curves after SG-MSC and SG-SNV preprocessing are shown in (d) and (f), respectively. It can be observed that the noise spikes are notably reduced, and the spacing between spectral lines is also significantly smaller. By preprocessing the near-infrared spectral data, the quality of the spectral data can be effectively enhanced, improving the accuracy of subsequent classification models.

### 3.3. Qualitative Detection of Coffee Adulteration

SVM (Support Vector Machine) is a supervised learning algorithm with strong capability in handling high-dimensional data, demonstrating excellent performance in classification tasks. The core idea of SVM is to find a hyperplane that separates the samples of different classes while maximizing the classification margin [29]. Therefore, SVM was chosen for the qualitative detection of coffee adulteration, specifically for distinguishing between the four types of adulterants in coffee, namely soybean, barley, chicory, and corn. To explore the most suitable data preprocessing method for qualitative adulteration detection in coffee, this study performed modeling on the raw spectral data as well as data preprocessed using the SG, SNV, MSC, SG-MSC, and SG-SNV methods. The results are shown in Table 2.

**Figure 3:**
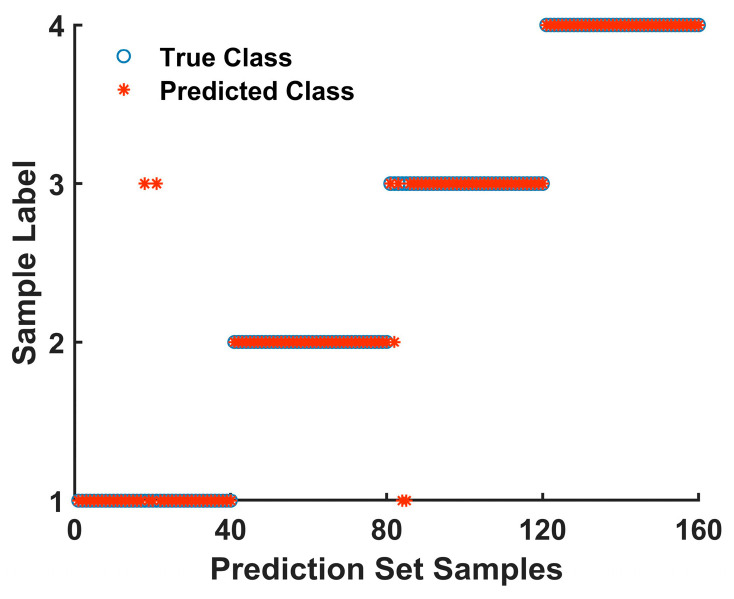
SG-MSC-SVM Classification Results for Coffee Adulteration Detection.

As shown in Table 2, the SVM model established using the raw spectral data achieved an accuracy of 93% for the qualitative detection of coffee adulteration. However, the models built with spectral data preprocessed by the five different methods showed further improvements in accuracy, all reaching over 95%. Although the adulterant was added to the coffee in varying proportions, a change in the adulterant ratio had little impact on the detection of the adulterant type. The model was able to accurately identify the type of adulterant present in the samples, indicating that the combination of near-infrared spectroscopy and the SVM algorithm can effectively detect the different types of materials adulterating coffee. Comparing the modeling results, it was found that the SG-MSC preprocessing method yielded the best model accuracy, as shown in Figure 3, with only four samples being misclassified. The classification accuracy reached 96.88%. Compared with the results of Chen Xiuming et al. [2], more adulterated species can be detected in this study, which has strong generalization ability and can adapt to different inputs and tasks. Therefore, SG-MSC-SVM was chosen as the model for the qualitative detection of coffee adulteration.

### 3.4. Qualitative and Quantitative Detection of Coffee Adulteration

#### 3.4.1. Spectral Data Preprocessing and Modeling

To address the complex detection problem that requires both the identification of the adulterant types in coffee and the determination of their adulteration ratios (qualitative and quantitative detection), this study employed three algorithms—SVM, BP neural network, and RF—on the data preprocessed using the following five methods: SG, SNV, MSC, SG-MSC, and SG-SNV. These models were compared with the results obtained from the raw spectral data, as shown in Table 3. Compared with Table 2, it can be observed that as the detection requirements increase, the overall model accuracy decreases. A comparison of the modeling results in Table 3 for different preprocessing methods and algorithms shows that the combination of SG-MSC preprocessing and the SVM model yields the best performance, with a classification accuracy of 83.13%. Therefore, for subsequent studies, the SG-MSC preprocessing method was chosen, and SVM was selected as the model for both the qualitative and quantitative detection of coffee adulteration.

#### 3.4.2. Feature Wavelength Selection of Spectral Data

To further improve the classification accuracy, it is necessary to perform feature wavelength selection on the spectral data preprocessed by SG-MSC. This process helps to eliminate redundant information in the near-infrared spectral data and retain the feature wavelengths that can effectively differentiate between the samples. In this study, the IWO algorithm and BChOA algorithm were employed for feature wavelength selection.

**Figure 4:**
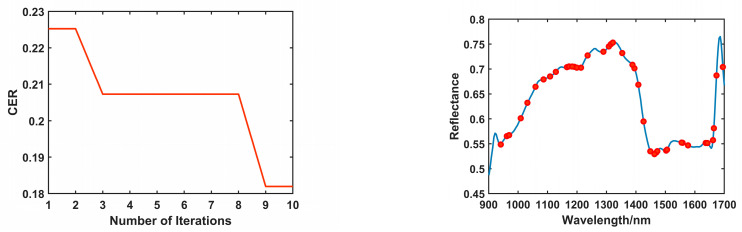
IWO feature extraction process (**left**) and results (**right**).

During the feature wavelength selection process using the IWO algorithm on the preprocessed full spectral data, the number of iterations was set to 10, with 15% of the data used as a validation set. The process of selecting feature wavelengths for the different coffee adulterant types using the IWO algorithm is illustrated in the left panel of Figure 4. As shown, with the increase in iteration number, the Classification Error Rate (CER) value gradually decreased and reached a local minimum during the fourth to eighth iteration. Subsequently, at the ninth iteration, the CER value reached its lowest point of 0.1819 and stabilized. At this point, a total of 40 feature wavelengths associated with the different coffee adulterant types were selected, and their distribution is shown in the right panel of Figure 4.

**Figure 5:**
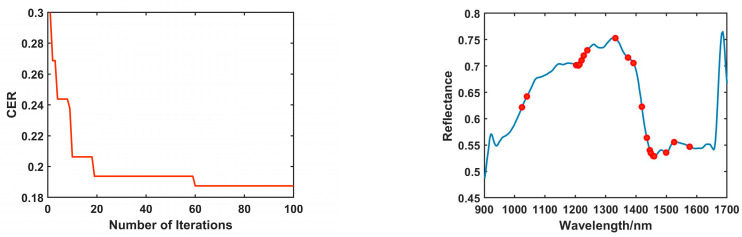
BChOA feature extraction process (**left**) and results (**right**).

During the feature wavelength selection process using the BChOA algorithm on the preprocessed full spectral data, the number of iterations was set to 100, with 20% of the data used as a validation set. The process of selecting feature wavelengths for the different coffee adulterant types using the BChOA algorithm is illustrated in the left panel of Figure 5. In the first 19 iterations, the Classification Error Rate (CER) value continuously decreased. However, at the 20th iteration, the CER value stabilized at 0.1937 and remained unchanged. This indicates that the feature variable combination obtained at this point was not the optimal solution. It was not until the 60th iteration that the CER value continued to decrease, reaching 0.1875, after which it remained stable. Finally, a total of 20 feature wavelengths related to the different coffee adulterant types were selected, and their distribution is shown in the right panel of Figure 5.

By comparing the feature wavelengths selected by the two feature selection algorithms shown in the right panels of Figure 4 and Figure 5, it can be observed that the feature wavelengths selected by the IWO algorithm are primarily distributed in the 970 nm, 1165–1213 nm, 1289–1394 nm, 1448–1576 nm, and 1637 nm regions. In contrast, the BChOA algorithm selected feature wavelengths primarily in the 1202–1239 nm, 1332 nm, and 1435–1525 nm regions. In these regions, the frequency bands around 900–1000 nm correspond to the third overtone of the CH, CH2, and CH3 groups, which can be attributed to the influence of the C-H groups in phenolic compounds [30]. The frequency band around 1150 nm corresponds to the second overtone of the C-H group, which is related to the presence of trigonelline and caffeine in coffee, as well as starch and lipid compounds such as fatty acids and triglycerides found in these materials. This frequency band also corresponds to the stretching vibration of C-O groups in starch [31,32]. The frequency bands around 1200–1300 nm are related to the stretching vibration of the C-O group, which is associated with alcohol compounds [31]. The frequency bands between 1350 and 1600 nm correspond to the first overtone of the O-H and N-H groups, which are associated with trigonelline, chlorogenic acid, and caffeine in coffee, as well as carbohydrates present in these materials. The vibrations around 1540 nm in this region are associated with the C-H stretching vibration in amide compounds [31]. To more directly compare the effectiveness of the two feature selection algorithms, this study constructed SVM models based on the feature wavelengths selected by each algorithm, and the results are shown in Table 4.

**Figure 6:**
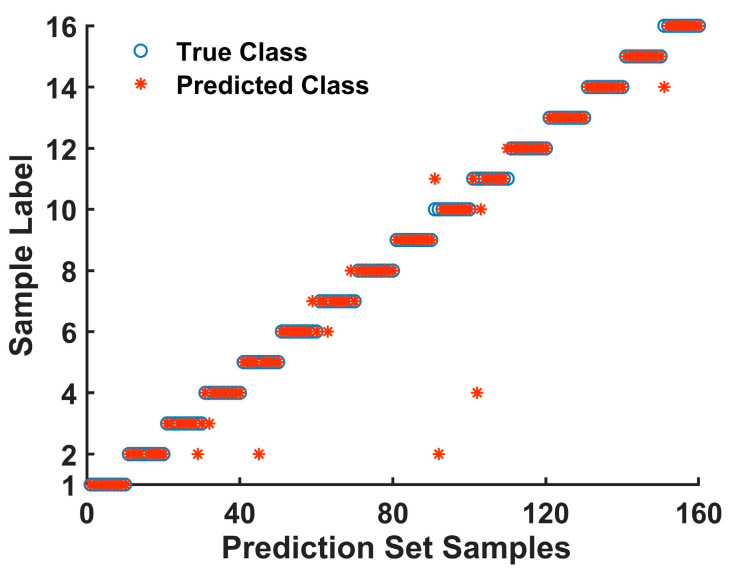
IWO-SVM Quantitative Classification Results for Coffee Adulteration Detection.

By comparing the results of the classification models built with the full-spectrum data, the feature wavelengths selected by the IWO algorithm, as well as those selected by the BChOA algorithm in Table 3, it is evident that the SVM models constructed using the feature wavelengths from both the IWO and BChOA algorithms achieved better results compared to the full-spectrum data. The dimensionality of the data was reduced by 82.46% and 91.23%, respectively, while the classification accuracy improved by 10.96% and 5.26%. This demonstrates that both feature wavelength selection methods effectively retain useful information that can characterize the differences between adulterated samples. These results suggest that feature wavelength selection can reduce model complexity while enhancing classification accuracy, which is consistent with the conclusions of Sun Jun et al. [33]. Among them, the model built using the feature wavelengths selected by the IWO algorithm achieved the best classification result, with an accuracy of 92.25%. In contrast, the model constructed using the wavelengths selected by the BChOA algorithm only reached an accuracy of 87.5%, indicating a less optimal outcome. This is because the BChOA algorithm selected only 20 feature wavelengths, which led to the loss of the effects of phenolic compounds in the 900–1000 nm region, the stretching vibrations of C-O and C-C groups in starch around 1150 nm, as well as the C-H groups in characteristic compounds in coffee, and the lack of the 1600 nm region, which corresponds to chlorogenic acid in coffee. Therefore, during the feature wavelength selection process, the BChOA algorithm missed some important wavelengths relevant to the qualitative and quantitative detection of coffee adulteration, which resulted in a slightly lower classification accuracy (by 4.75%) compared to the model built with the feature wavelengths selected by the IWO algorithm. As a result, IWO-SVM was selected as the model for coffee adulteration detection, with the classification results shown in Figure 6. The IWO-SVM model only needs 40 characteristic wavelengths to realize the qualitative and quantitative detection of coffee adulteration, which makes up for the limitation of the qualitative or quantitative analyses in previous studies. Compared with the traditional methods (chromatography, mass spectrometry), the model can not only quickly and nondestructively determine whether coffee samples are adulterated (qualitative detection) but also quantitatively analyze the proportion of adulterants.

## 4. Conclusions

This study proposes a rapid, non-destructive detection method for coffee adulteration based on portable near-infrared spectroscopy technology and establishes both the qualitative and quantitative detection models for coffee adulteration. The results demonstrate that preprocessing near-infrared spectral data can significantly improve model performance, with the combination of SG-MSC yielding better results than the SG, MSC, SNV, SG-SNV, and raw spectral data. In the identification of adulterant types in coffee, the SVM model achieved a classification accuracy of 96.88%, a precision of 96.86%, and a specificity of 98.96%, with only four samples misclassified. When determining both the type and corresponding proportion of adulterants, the SVM model performed better than BP and RF using different preprocessing methods. Feature wavelengths selected by the IWO algorithm were more effective in representing the differences between the samples. Compared to the full-spectrum data and wavelengths selected by the BChOA algorithm, the model based on the IWO algorithm showed a significant advantage in accuracy. The IWO-SVM model achieved a classification accuracy of 92.25%, precision of 92.61%, and specificity of 99.42%. Thus, the method proposed in this study enables the rapid, non-destructive detection of coffee adulteration, providing a theoretical foundation for the further development of portable coffee adulteration detection devices. It also addresses the time-consuming and costly issues of the traditional detection methods, while offering valuable insights for the rapid, non-destructive detection of adulteration in other food products.

Although this study has yielded positive results, there is still room for further research and optimization. The current study is based solely on Arabica coffee beans, which, while cultivated worldwide, do not encompass all possible real-world scenarios or different varieties. Therefore, future research could involve collecting data from a broader range of coffee bean varieties, particularly spectral data under different adulteration conditions, to increase the diversity of the data and enhance the model’s generalization ability. Additionally, future work could consider integrating the model into a smartphone app, connecting it via Bluetooth to portable near-infrared spectrometers for on-site, or real-time coffee adulteration detection. This approach would not only improve quality control efficiency in the coffee industry but also provide consumers with convenient and reliable tools for ensuring product authenticity and safety. Moreover, the application of this method is not limited to coffee adulteration detection but can also be extended to other food sectors, such as dairy products, juices, and grains. This would help improve the accuracy and efficiency of food quality control, product traceability, and supply chain management, offering more effective and reliable technological support for food safety.

## Figures and Tables

**Table 1:** Basic Information of Adulterants.

Adulterants	Origin	Price (CNY/kg)	Temperature (°C)	Time (min)
Soybean	Harbin, Heilongjiang Province, China	11	190	45
Barley	Harbin, Heilongjiang Province, China	9	180	40
Chicory	Changchun, Jilin Province, China	35	170	35
Corn	Handan, Hebei Province, China	7.92	180	45

**Table 2:** Modeling Results of Spectral Data Based on Different Preprocessing Methods.

Preprocessing Method	Training Set			Prediction Set		
	Accuracy/%	Precision	Specificity	Accuracy/%	Precision	Specificity
RAW	98.13	98.13	99.38	93.75	93.78	97.92
MSC	96.72	96.75	98.91	96.25	96.48	98.75
SG	97.81	97.81	99.27	96.25	96.39	98.75
SNV	96.09	96.08	98.70	95.63	95.79	98.54
SG-SNV	98.75	98.75	99.58	95.01	95.07	98.33
SG-MSC	97.03	97.03	99.01	96.88	96.86	98.96

**Table 3:** Modeling Results of Spectral Data Based on Different Preprocessing Methods.

Model	Preprocessing Method	Training Set			Prediction Set		
		Accuracy/%	Precision	Specificity	Accuracy/%	Precision	Specificity
SVM	RAW	73.43	74.13	98.23	69.38	70.86	97.96
	MSC	67.68	69.53	97.85	65.42	67.30	97.69
	SG	75	75.18	98.33	74.38	75.71	98.29
	SNV	76.41	77.79	97.88	68.13	97.88	72.24
	SG-SNV	83.44	84.36	98.90	77.5	78.10	98.5
	SG-MSC	86.09	86.92	99.07	83.13	84.26	98.88
BP	RAW	72.03	63.83	97.17	72.5	58.33	96.67
	MSC	82.97	82.93	98.83	77.5	75	98
	SG	70.94	56.86	96.33	71.88	58.82	95.33
	SNV	78.91	63.64	96.67	77.5	55.56	94.67
	SG-SNV	77.19	69.57	97.67	75.63	58.33	96.67
	SG-MSC	85.31	88.37	99.17	81.25	66.67	96.67
RF	RAW	74.53	63.41	97.5	66.25	66.67	98
	MSC	84.38	86.37	99	78.13	63.64	97.33
	SG	76.56	71.43	98	70.13	63.33	96
	SNV	79.84	68.09	97.5	76.25	63.64	97.33
	SG-SNV	81.41	75.56	98.17	76.88	72.73	98
	SG-MSC	82.34	75.56	98.17	76.88	60	97.33

**Table 4:** SVM Model Results Based on Different Feature Extraction Algorithms.

Feature Selection	Data Dimensions	Training Set			Prediction Set		
		Accuracy/%	Precision	Specificity	Accuracy/%	Precision	Specificity
FULL	228	86.09	86.92	99.07	83.13	84.26	98.88
IWO	40	96.88	96.93	99.80	92.25	92.61	99.42
BChOA	20	89.06	89.29	99.27	87.5	88.78	99.17

## Data Availability

The original contributions presented in the study are included in the article. Further inquiries can be directed to the corresponding author.
